# Risk factors for local recurrence following marginal mandibulectomy in gingival cancer

**DOI:** 10.1038/s41598-024-77239-3

**Published:** 2024-11-01

**Authors:** Olof Nilsson, Mathias von Beckerath, Johan Knutsson, Anders Magnuson, Fredrik J. Landström, Rusana Bark

**Affiliations:** 1 Department of Otolaryngology, Örebro University Hospital, Södra Grev Rosengatan, 701 85 Örebro, Sweden; 2 School of Medical Sciences, Faculty of Medicine and Health, Örebro University, Örebro, Sweden; 3 Medical Unit Head Neck Lung and Skin Cancer, Department of Head and Neck Surgery, Karolinska University Hospital, Stockholm, Sweden; 4 Department of Clinical Sciences Intervention and Technology, Division of Ear Nose and Throat Diseases, Karolinska Institute, Stockholm, Sweden; 5 Department of Otolaryngology, Vasteras Hospital, Vasteras, Sweden; 6 Centre for Clinical Research, Vastmanland Hospital, Region Vastmanland-Uppsala University, Vasteras, Sweden; 7 Clinical Epidemiology and Biostatistics, School of Medical Sciences, Faculty of Medicine and Health, Örebro University, Örebro, Sweden

**Keywords:** Marginal mandibulectomy, Gingival cancer, Local recurrence, Gingival neoplasm, Cancer, Surgical oncology

## Abstract

Surgery is the first line of treatment in gingival cancers of the mandible, and bone resection is necessary in the majority of cases. In the less extensive surgical option, marginal mandibulectomy (MM), the mandibular base is preserved. In contrast, in a segmental mandibulectomy (SM) the mandible is divided and the continuity is not preserved. If MM can be performed with comparable oncological results to SM, it is the preferred method. The aim of the present study was to identify preoperative predictors for local recurrence (LR), to support the selection of candidates for MM. Outcome measures were local recurrence free survival (LRFS) and disease specific survival (DSS). 67 patients treated with MM between 2008 and 2021 were included. Cox regression analyses of LR with hazard ratios and adjustments for postoperative radiotherapy, pathological T-stage (pT) and soft tissue margins were performed. 5-years LRFS was 63% (95% CI 46.9–75.5) and DSS 80.6% (95% CI 64.7–89.9). In conclusion we found that edentulous patients, more advanced pT-stage and positive soft tissue margins had increased risk for LR. Future studies of the correlation between cT and pT would be important to provide more robust preoperative support in the selection between MM and SM.

## Introduction

Surgery is the first line of treatment in gingival cancer of the mandible, and bone resection is necessary in the majority of cases. Marginal mandibulectomy (MM) preserves the mandibular base and thereby also the continuity of the mandible. In contrast, in a segmental mandibulectomy (SM) the mandible is divided and the continuity is not preserved. Furthermore, MM requires a less advanced reconstruction than SM to achieve an acceptable functional and esthetical outcome. For SM, reconstruction with a bony free flap is the best choice to repair the defect and to restore the continuity of the mandible^1,2^. Although the esthetical and functional results after a successful bony free flap reconstruction are very good, the surgery is more time consuming, has higher peri- and postoperative risks for complications and is more expensive. Moreover, older and frail patients might not tolerate extensive surgery and the options available include the use of a reconstruction plate or no restoration of the continuity at all. Therefore, if MM can be performed with comparable oncological results to SM, it is the preferred method.

Knowledge about the bone invasion of the mandible is crucial in order to determine whether a MM is oncologically safe or not. It is well established that tumours invade the mandible at the point of abutment (the point of contact between the tumour and the bone)^3^. Clinical assessment, computed tomography and/or magnetic resonance imaging are used to evaluate bone invasion of the mandible. Despite improved radiological imaging techniques, it is not possible to detect microscopic invasion and the clinical T-stage (cT) classification can be difficult. Superficial, erosive bone invasion is not sufficient for cT4a classification of the tumour and does not preclude a MM. For early oral cancers, including cT1-T2 gingival cancers, MM is now an established surgical treatment option^4,5^.

There are certain scenarios where SM is the method of choice, including obvious bone invasion into the bone marrow or the mandibular canal. In addition, in edentulous patients the height of the mandible often does not permit a MM since at least 10 mm needs to be preserved to decrease the risk of a fracture^6^. The use of MM in radiated patients or patients with large soft tissue tumours is controversial^4^.

However, there is a lack of convincing evidence for the proper selection of surgical method and predictors for local recurrence and survival. Most studies include all subsites of oral squamous cell carcinoma (OSCC), and are not specific for gingival cancer. Since MM is considered a surgical treatment option for selected cT1-T2 tumours and SM for the more advanced tumours, the methods cannot be directly compared in the existing, exclusively retrospective studies.

The aim of the present study was to identify predictors that can support the selection between marginal and segmental mandibulectomy. We specifically searched for preoperative factors resulting in poor outcome in patients undergoing MM. Our primary outcome measure was local recurrence free survival (LRFS) and secondary outcome was disease specific survival (DSS).

## Method

This was a retrospective cohort study of patients with gingival cancer of the mandible. The study was approved by the Swedish Ethical Review Authority (2020–06118). Obtaining informed consent was waived by the same authority due to the retrospective nature of the study. The treatment options offered to the patients are gold standard for gingival cancer of the mandible, in adherence to Swedish and international guidelines for head and neck cancer^7,8^.

**Fig. 1:**
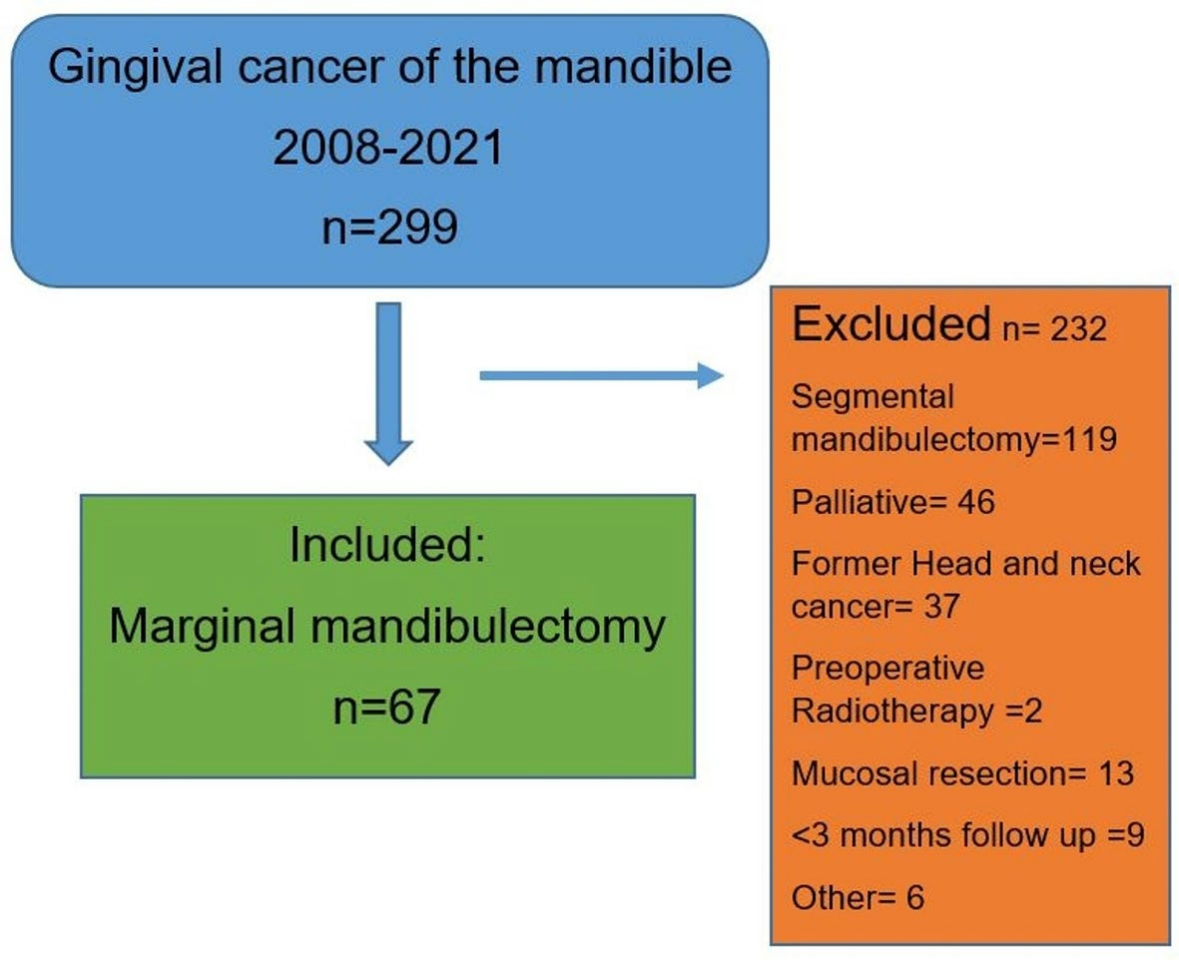
Flowchart with inclusion and exclusion criteria.

All patients with ICD-10 code C03.1 (malignant neoplasm of the lower gum) treated between the years 2008 and 2021 at two Swedish head and neck cancer centres, Stockholm and Örebro, were retrieved from the Swedish Head and Neck Cancer Register (SweHNCR) and the Regional Head and Neck Register in Örebro, and considered for inclusion (*n* = 299)^9,10^. A review of the medical records was performed. Tumours were staged according to the TNM clinical staging system^11^. Exclusion criteria presented in Fig. 1 were: segmental mandibulectomy, palliative intent, former head and neck cancer, preoperative radiotherapy, mucosal resection without bone resection or follow-up time less than three months. Extracted data included, but was not limited to, age, gender, histopathology reports, clinical and pathological TNM status, as well as treatment modality, local recurrence (LR) and overall and disease specific survival (OS/DSS respectively).

For the primary outcome measure LRFS, time to event (LR) was calculated from 3 months after the date of surgery (T0), to take into account the time of eventual postoperative radiotherapy (PORT) and residual tumours. Patients were censored in case of death, second primary or loss to follow-up (maximum follow-up time 5 years from T0). Cox regression analyses with hazard ratios (HR) and adjustments for the potential confounders PORT, pathological T-stage (pT) and soft tissue margins were performed.

In the same way DSS was calculated from 3 months after the date of surgery, to the event death with tumours. Patients were censored if they died without tumours, had a second primary or were lost to follow-up. Kaplan-Meier curve was used for DSS; however, since only nine events appeared, no regression analysis was performed.

Variables in the regression analyses were grouped into those known before and after surgery, in accordance with the clinical situation where the surgical treatment decision is based on preoperative variables. Gingival cancer grow close to the teeth and there have been concerns that these tumours easily spread into the bone marrow through the tooth sockets. Therefore, we included the variables dental status and tumour location (separating tumours reaching or not reaching the teeth). Soft tissue margins with tumours at the resection border in the pathology report were defined as positive margins, whereas soft tissue margins less than 5 mm were defined as close margins. The variable tumour growth in bone marrow involved invasion into the bone marrow only, or into the mandibular canal as well.

Overall a p-value < 0.05 was considered to be statistically significant. Analyses were performed using SPSS version 29.0 and STATA release 17.

## Results

**Fig. 2:**
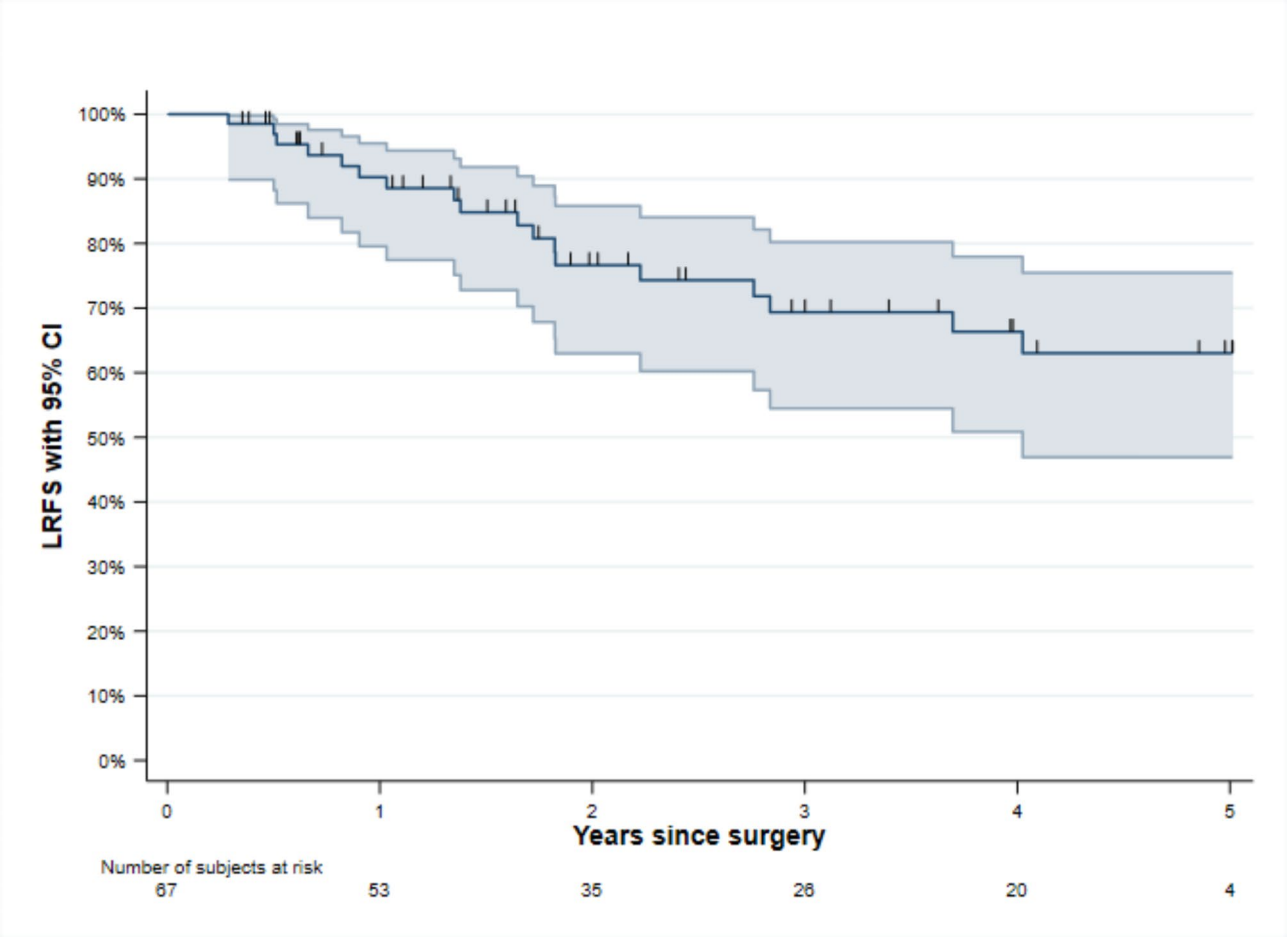
Kaplan-Meier Curve of 5-years local recurrence free survival (LRFS) with 95% CI.

There were 67 patients treated with MM included in the study, 22 patients from Örebro and 45 from Stockholm, see also flow chart in Fig. 1. Demographics of the cohort are provided in Table 1. In total there were 18 (26.8%) LR and 5-years LRFS was 63.0% (95% CI 46.9–75.5), Fig. 2. Median follow-up time was 22.9 months (IQR 10.2–56.6). Tumours classified as pT3 had significantly increased hazard ratio (HR) for LR in the unadjusted and adjusted regression analyses, 8.61 (95% CI 1.43–51.9) and 6.64 (95% CI 1.05–41.9), respectively. For pT4a tumours the unadjusted HR was 4.86 (95% CI 1.01–23.4), while the adjusted HR was not significant.

**Table 1:** Patient demographics.

	Marginal mandibulectomy (*n* = 67)
**Variables known before surgery**	
Age, median (IQR)	75 (64–80)
Sex, n (%)	
Female	36 (53.7)
Male	31 (46.3)
Smoking, n (%)	
Non-smoking	29 of 63 (46.0)
Former smoking	16 of 63 (25.4)
Current smoking	18 of 63 (28.6)
WHO performance status, n (%)	
WHO 0	57 of 65 (87.7)
WHO 1–3	8 of 65 (12.3)
cT stage, n (%)	
T1	29 (43.3)
T2	26 (38.8)
T3	4 (6.0)
T4a	8 (11.9)
cN stage, n (%)	
N0	56 (83.6)
N1	6 (9.0)
N2b/c	5 (7.5)
Tumour location, n (%)	
Tumour reaches the teeth	61 (91.0)
Tumour does not reach the teeth	6 (9.0)
Dental status in tumour region, n (%)	
Healthy teeth	41 (61.2)
Loose/extracted tooth	18 (26.9)
Edentulous	8 (11.9)
**Variables known after surgery**	
pT stage, n (%)	
T1	25 (37.3)
T2	18 (26.9)
T3	6 (9.0)
T4a	18 (26.9)
Soft tissue margin, n (%)	
Negative margin	20 (29.8)
Close marginal	34 (50.8)
Positive margin	13 (19.4)
Bone invasion pathology report (*n* = 65), n (%)	
No bone invasion	41 (63.1)
Superficial or bone invasion ND^1^	12 (18.5)
Tumour growth in bone marrow, n (%)	12 (18.5)
Postoperative Radiotherapy, n (%)	37 (55.2)

The increased HR observed in higher pT classification did not significantly correspond to the clinical T-stage (cT) classification, see also Table 2. In tumours staged as cT1 that in fact were pT2-pT4a, a LR was detected in four of the seven cases (57.1%). In total there were five LR seen in cT1 tumours, four of which were understaged. For the nine understaged cT2 tumours, three (33.3%) LR were observed. There were eight LR altogether in cT2 tumours, three of them understaged. The rates for LR were lower for both cT1 and pT1 tumours compared to tumours with higher T classifications, Table 2.

**Table 2:** Five years local recurrence risk evaluated with Cox regression, follow-up starts 3 months after surgery, *n* = 67 and 18 local recurrences.

Variables known before surgery	Events	Rates	Unadjusted	Adjusted^a^
			HR (95% CI)	aHR (95% CI)
Age as spline				
65			Ref	Ref
75			1.12 (0.42–3.01)	1.15 (0.39–3.37)
85			1.04 (0.28–3.93)	1.59 (0.34–7.43)
Sex				
Men	9	108.2	Ref	Ref
Women	9	95.5	0.85 (0.34–2.15)	0.71 (0.27–1.86)
Smoking (*n* = 63)				
Non-smoker	9	95.3	Ref	Ref
Current	4	97.8	1.02 (0.31–3.32)	0.58 (0.14–2.40)
Former	4	121.2	1.22 (0.37–4.05)	0.55 (0.11–2.72)
WHO status (*n* = 65)				
WHO 0	15	95.0	Ref	Ref
WHO 1–3	3	313.6	3.30 (0.94–11.5)	4.40 (0.93–20.8)
cT-stage				
T1	5	64.0	Ref	Ref
T2	8	122.0	2.01 (0.66–6.16)	1.40 (0.42–4.62)
T3	1	103.5	1.48 (0.17–12.7)	2.03 (0.23-18.0)
T4a	4	166.7	2.52 (0.67–9.39)	2.09 (0.50–8.64)
cN-stage				
N0	15	100.0	Ref	Ref
N+	3	109.9	1.09 (0.31–3.75)	0.97 (0.23–4.01)
Tumour location				
Do not reach the teeth	1	59.8	Ref	Ref
Reaches the teeth	17	105.8	1.91 (0.25–14.4)	1.22 (0.12–12.9)
Dental status in tumour region				
Healthy	9	76.4	Ref	Ref
Loose/extracted tooth	3	71.3	0.88 (0.24–3.27)	1.18 (0.31–4.56)
Edentulous	6	344.3	4.07 (1.43–11.6)	1.87 (0.58–5.99)
**Variables** **known after surgery**				
pT stage				
T1	2	28.9	Ref	Ref
T2	6	132.2	4.71 (0.95–23.4)	2.94 (0.52–16.7)
T3	3	258.6	8.61 (1.43–51.9)	6.64 (1.05–41.9)
T4a	7	137.0	4.86 (1.01–23.4)	2.17 (0.40–12.0)
Soft tissue margin				
Negative	2	29.8	Ref	Ref
Close	9	107.0	3.45 (0.74-16.0)	2.81 (0.55–14.4)
Positive	7	267.4	8.82 (1.83–42.6)	5.85 (1.02–33.6)
Tumour growth in bone marrow				
No	13	87.2	Ref	Ref
Yes	5	176.6	2.03 (0.72–5.71)	1.64 (0.31–8.78)
Postop radiotherapy				
No	3	39.6	Ref	Ref
Yes	15	147.6	3.83 (1.11–13.3)	1.68 (0.39–7.35)

^a^

Adjusted for PORT (postoperative radiotherapy), pT stage and Soft tissue margin.

Crude rates per 1000 person-years; HR Hazard ratios; aHR adjusted Hazard ratios; CI Confidence intervals.

Positive soft tissue margins were a strong predictor for LR in the unadjusted and adjusted analyses, 8.82 (95% CI 1.82–42.6) and 5.85 (95% CI 1.02–33.6) respectively.

LR was detected in 6 of the 8 edentulous patients (75%). The unadjusted HR for edentulous patients was 4.22 (95% CI 1.57–11.3), adjusted HR not significant.

**Fig. 3:**
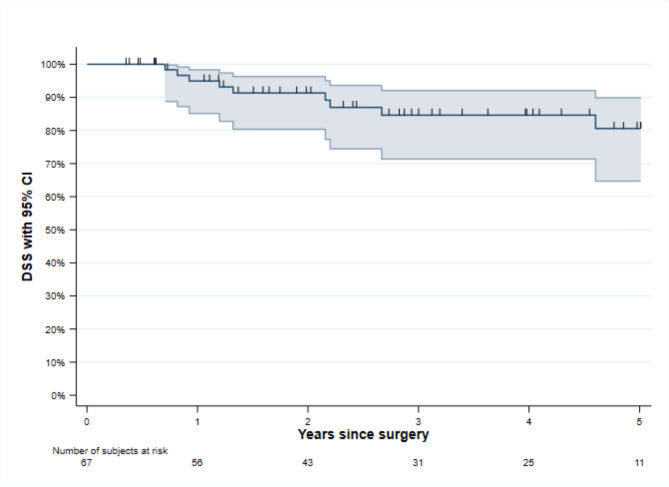
Kaplan-Meier curve of 5-years disease specific survival (DSS) with 95% CI.

DSS after 5-years was 80.6% (95% CI 64.7–89.9) and is presented in Fig. 3. There were nine patients dead with tumours, and among them seven had LR detected. Even though a LR is a serious event, 10 patients with LR were censored for follow-up in the survival analyses (meaning they did not have the event death with tumours). Median follow-up time was 31.3 months (IQR 12.9–57.0).

The majority of the patients had no flap reconstruction (*n* = 43, 67.2%) or local flap reconstruction (*n* = 13, 20.3%).

## Discussion

The present study specifically included patients with gingival cancer of the mandible, treated with marginal mandibulectomy. The aim was to identify preoperative variables, known before surgical treatment to support the selection between MM and SM.

The main finding in this study was an increased risk for LR in higher pT-stage, especially pT3-pT4a. On the contrary, the cT-stage did not significantly correlate with LR. This could be due to misclassification, since more LR appeared in understaged cT1 tumours. Moreover, edentulous patients had a high frequency of LR, signalling caution when considering the use of MM for these patients. In addition, positive soft tissue margins were a strong predictor for LR. This is well known, but important to emphasize since insufficient soft tissue margins are common in OSCC^4,12,13^ and there is room for improvement.

Although many studies have examined predictors of the oncological outcome of MM in oral cavity cancers^4,12,14–21^, there is a lack of knowledge about preoperative variables. Perhaps the increased risk for LR observed in edentulous patients, could be explained by a high degree of positive soft tissue margins (37.5%) and a higher T-stage (no cases of pT1 were edentulous). Nonetheless, dental status is known before surgery, as opposed to soft tissue margins and pT-stage.

Similarly, the clinical and radiological assessments of the tumours used in the cT classification are known before the decision of the extent of the surgical treatment is made. In our study the misclassifications of the cT compared to the pT were quite high, 17.9% cT3-cT4a vs. 35.8% pT3-T4a. The LR and LRFS in the present study were 26.8% and 63% respectively, as compared to the reported LR of 5.6–26.4%, and LRFS of 74.6–85.0% in the literature^4,12,14,18–22^. Still, the DSS in the present study was comparable to the results seen in several other studies^4,16,19,22^, indicating that salvage treatment was successful for some of the patients with LR. Further, direct comparison is difficult due to several factors: most studies encompass all subsites of OSCC, the degree of bone invasion varies, indications for MM and PORT differ. The surprisingly high amount of cT3-T4a as well as pT3-T4a in our cohort is probably one explanation for the relatively high degree of LR observed. Improved classification of cT, aligned with better agreement to pT, would be important to decrease the risk of undertreatment of understaged tumours. Clinical and radiological assessments determine the cT-stage classification, but analyses of these factors are outside the scope of this study.

Additionally, soft tissue margins are well known to influence the risk for LR, and studies with clear margins and indications for MM restricted to cT1-T2 seem to have less LR^14,18,20^. Whether tumours with bone invasion or insufficient bone margins portend increased risk for LR is more controversial. Bone invasion into the bone marrow and/or mandibular canal appears to be important for DSS and risk for distant metastasis^16–19^, and as pointed out before the recommended surgical resection is SM. The size of the tumours is an established risk factor incorporated in the TNM classification^23^, and actually more important than bone invasion, at least for tumours smaller than 4 cm^12,16,17^. Several studies have suggested a modified T-staging system within the TNM classification, to provide better prognostic information. In this system, the tumours would first be classified as cT1-T3 based on size, and then upstaged by one T-stage in cases of medullary bone invasion^12,16,18^. Bone invasion of the mandibular canal would always be classified as cT4a.

## Limitations and future studies

The retrospective and observational design are obvious limitations in the present study. This involves the risk of information bias and potential inconsistencies in tumour classification and indications for MM and PORT between different time periods and treatment centers. Differing definitions of bone invasion could be one explanation for the misclassification of cT compared to pT observed in the present study study. Another limitation is that the confidence intervals are generally wide due to low power, a result of the small sample size and the low number of events in the analyses of LRFS and DSS. Because gingival cancer of the mandible is a rare disease, the cohort in the present study still remains one of the larger ones on the subject and therefore contributes to the existing evidence. Also, this study benefits from analysing MM specifically, and thereby avoiding the potential bias of mixing results from treatment by MM and SM, but the above mentioned weaknesses prevent the drawing of strong conclusions.

The most established preoperative risk factor for LR is the cT-stage. Optimising the T-stage classification is important to accurately select candidates for MM. We believe future studies would benefit from specifically including gingival cancers and, if possible, have a prospective design to reduce the risk of bias. Furthermore, future studies should consider evaluating the modified T-stage classification first suggested by Ebrahimi et al.^16^. Determining the clinically significant soft tissue margin in mm would be important, as close margins have a broad definition (0.01–4.9 mm). This could help guide decisions about adjuvant treatment.

## Conclusions

Edentulous patients had an increased risk for LR after MM for gingival cancers of the mandible. More advanced pT-stage and positive soft tissue margins also correlated to more LR, though they are not preoperatively known factors. Future studies of the correlation between cT and pT would be important to provide more robust preoperative support in the selection between MM and SM.

## Data Availability

The data that support the findings of this study are not openly available, but pseudonymised data can be retrieved from the corresponding author upon reasonable request. Data are located in controlled access data storage at Örebro University Hospital.

## References

[1] Mücke, T. et al. Comparison of outcome of microvascular bony head and neck reconstructions using the fibular free flap and the iliac crest flap. *Br. J. Oral Maxillofac. Surg.*** 51** (6), 514–519 (2013). 23399107 10.1016/j.bjoms.2013.01.007

[2] Wilkman, T., Husso, A. & Lassus, P. Clinical comparison of scapular, fibular, and Iliac crest osseal free flaps in maxillofacial reconstructions. *Scand. J. Surg.*** 108** (1), 76–82 (2019). 29732952 10.1177/1457496918772365

[3] Brown, J. S. et al. Patterns of invasion and routes of tumor entry into the mandible by oral squamous cell carcinoma. *Head Neck*. **24** (4), 370–383 (2002). 11933179 10.1002/hed.10062

[4] Petrovic, I. et al. Influence of bone invasion on outcomes after marginal mandibulectomy in squamous cell carcinoma of the oral cavity. *J. Craniomaxillofac. Surg.*** 45** (2), 252–257 (2017). 28011180 10.1016/j.jcms.2016.11.017 PMC5293664

[5] Rao, L. P., Shukla, M., Sharma, V. & Pandey, M. Mandibular conservation in oral cancer. *Surg. Oncol.*** 21** (2), 109–118 (2012). 21856149 10.1016/j.suronc.2011.06.003

[6] Barttelbort, S. W. & Ariyan, S. Mandible preservation with oral cavity carcinoma: Rim mandibulectomy versus sagittal mandibulectomy. *Am. J. Surg.*** 166** (4), 411–415 (1993). 8214304 10.1016/s0002-9610(05)80344-7

[7] Regionalt cancercentrum. Nationellt vårdprogram Huvud- och Halscancer 2023. https://kunskapsbanken.cancercentrum.se/diagnoser/huvud-och-halscancer/vardprogram/. Accessed 2024 Mars 16.

[8] NCCN Guidelines Head and Neck Cancers. 2024. Version 4.2024. https://www.nccn.org/professionals/physician_gls/pdf/head-and-neck.pdf. Accessed 2024 Sept 26.

[9] Swedish Head and Neck Cancer Register (SweHNCR). https://www.cancercentrum.se/samverkan/vara-uppdrag/kunskapsstyrning/kvalitetsregister/om-inca/. Accessed 2024 Oct 7.

[10] Head and Neck Register in Örebro. Region Örebro County.

[11] Sobin, L. H., Gospodarowicz, M. K. & Wittekind, C. *TNM Classification of Malignant Tumours* (Wiley-Blackwell, 2009).

[12] Fried, D. et al. Prognostic significance of bone invasion for oral cavity squamous cell carcinoma considered T1/T2 by American joint committee on cancer size criteria. *Head Neck*. **36** (6), 776–781 (2014). 23616341 10.1002/hed.23367

[13] Tasche, K. K., Buchakjian, M. R., Pagedar, N. A. & Sperry, S. M. Definition of close margin in oral cancer surgery and association of margin distance with local recurrence rate. *JAMA Otolaryngol.- Head Neck Surg.*** 143** (12), 1166–1172 (2017). 28445581 10.1001/jamaoto.2017.0548 PMC5824301

[14] Du, W., Fang, Q., Wu, Y., Wu, J. & Zhang, X. Oncologic outcome of marginal mandibulectomy in squamous cell carcinoma of the lower gingiva. *BMC Cancer*. **19** (1), 775 (2019). 31387576 10.1186/s12885-019-5999-0 PMC6683491

[15] Ebrahimi, A. et al. Primary tumor staging for oral cancer and a proposed modification incorporating depth of invasion: An international multicenter retrospective study. *JAMA Otolaryngol.-Head Neck Surg.*** 140** (12), 1138–1148 (2014). 25075712 10.1001/jamaoto.2014.1548

[16] Ebrahimi, A., Murali, R., Gao, K., Elliott, M. S. & Clark, J. R. The prognostic and staging implications of bone invasion in oral squamous cell carcinoma. *Cancer*. **117** (19), 4460–4467 (2011). 21437887 10.1002/cncr.26032

[17] Fives, C. et al. Impact of mandibular invasion on prognosis in oral squamous cell carcinoma four centimeters or less in size. *Laryngoscope*. **127** (4), 849–854 (2017). 27481484 10.1002/lary.26211

[18] Okura, M. et al. Prognostic and staging implications of mandibular canal invasion in lower gingival squamous cell carcinoma. *Cancer Med.*** 5** (12), 3378–3385 (2016). 27758080 10.1002/cam4.899 PMC5224841

[19] Patel, R. S. et al. The prognostic impact of extent of bone invasion and extent of bone resection in oral carcinoma. *Laryngoscope*. **118** (5), 780–785 (2008). 18300706 10.1097/MLG.0b013e31816422bb

[20] Suresh, S. et al. Doing as little as possible and as much as necessary’ - Oncological efficacy of marginal mandibulectomy in resection of oral cavity cancers. *Oral Oncol.*** 95**, 91–94 (2019). 31345400 10.1016/j.oraloncology.2019.05.026

[21] Werning, J. W., Byers, R. M., Novas, M. A. & Roberts, D. Preoperative assessment for and outcomes of mandibular conservation surgery. *Head Neck*. **23** (12), 1024–1030 (2001). 11774386 10.1002/hed.10031

[22] Shaw, R. J. et al. The influence of the pattern of mandibular invasion on recurrence and survival in oral squamous cell carcinoma. *Head Neck*. **26** (10), 861–869 (2004). 15390204 10.1002/hed.20036

[23] Brierley, J., Gospodarowicz, M. K. & Wittekind, C. *TNM Classification of Malignant Tumours* (Chichester, 2017).

